# A dataset of Antarctic ecosystems in ice-free lands: classification, descriptions, and maps

**DOI:** 10.1038/s41597-025-04424-y

**Published:** 2025-01-22

**Authors:** Anikó B. Tóth, Aleks Terauds, Steven L. Chown, Kevin A. Hughes, Peter Convey, Dominic A. Hodgson, Don A. Cowan, John Gibson, Rachel I. Leihy, Nicholas J. Murray, Sharon A. Robinson, Justine D. Shaw, Jonathan S. Stark, Mark I. Stevens, John van den Hoff, Jane Wasley, David A. Keith

**Affiliations:** 1 Centre for Ecosystem Science, School of Biological, Earth, and Environmental Sciences, University of New South Wales, Sydney, NSW 2052 Australia; 2 UNSW Data Science Hub, University of New South Wales, Sydney, NSW 2052 Australia; 3 Australian Antarctic Division, Department of Climate Change, Energy, the Environment and Water, 203 Channel Highway, Kingston, Tasmania 7050 Australia; 4 Securing Antarctica’s Environmental Future, School of Biological Sciences, Monash University, Victoria, 3800 Australia; 5 British Antarctic Survey, Natural Environment Research Council, High Cross, Madingley Road, Cambridge, CB3 0ET UK; 6 Department of Zoology, University of Johannesburg, Auckland Park, 2006 South Africa; 7 Millennium Institute Biodiversity of Antarctic and Subantarctic Ecosystems (BASE), Santiago, Chile; 8 Centre for Microbial Ecology and Genomics, Department of Biochemistry, Genetics and Microbiology, University of Pretoria, Pretoria, 0002 South Africa; 9 College of Science and Engineering, James Cook University, Townsville, Queensland 4811 Australia; 10 Environmental Futures, University of Wollongong, Wollongong, NSW 2522 Australia; 11 Securing Antarctica’s Environmental Future, University of Wollongong, Wollongong, NSW 2522 Australia; 12 Securing Antarctica’s Environmental Future, School of Biology and Environmental Science, Queensland University of Technology, Brisbane, Queensland 4001 Australia; 13 Securing Antarctica’s Environmental Future, Earth and Biological Sciences, South Australian Museum, North Terrace, Adelaide, SA 5000 Australia; 14 School of Biological Sciences, University of Adelaide, Adelaide, SA 5005 Australia

**Keywords:** Biodiversity, Conservation biology

## Abstract

Antarctica, Earth’s least understood and most remote continent, is threatened by human disturbances and climate-related changes, underscoring the imperative for biodiversity inventories to inform conservation. Antarctic ecosystems support unique species and genetic diversity, deliver essential ecosystem services and contribute to planetary stability. We present Antarctica’s first comprehensive ecosystem classification and map of ice-free lands, which host most of the continent’s biodiversity. We used latent variables in factor analyses to partition continental-scale abiotic variation, then biotic variation represented in spatial models, and finally recognised regional-scale variation among biogeographic units. This produced a spatially explicit hierarchical classification with nine Major Environment Units (Tier 1), 33 Habitat Complexes (Tier 2) and 269 Bioregional Ecosystem Types (Tier 3) mapped at 100 m resolution and aligned with ‘level 4’ of the IUCN Global Ecosystem Typology. This comprehensive ecosystem inventory provides foundational data to inform protected area designation under the Antarctic Treaty’s Environmental Protocol and track risks to Antarctic ecosystems. Its tiered structure and workflow accommodate data scarcity and facilitate updates, promoting robustness as knowledge builds.

## Background & Summary

Antarctica is one of the largest wilderness areas on Earth^1^. It has a unique system of governance (the Antarctic Treaty system; www.ats.aq), with specific guidance on how it should be managed and protected through the Protocol on Environmental Protection to the Antarctic Treaty (commonly known as the Environmental Protocol or Madrid Protocol^2^). Although permanently ice-free areas are home to most of the continent’s known terrestrial biodiversity, these rare and isolated systems cover only 0.2–0.5% of the continent^3,4^. Extremes of photoperiod and cold, distinct weather processes such as katabatic winds, and the gradients created by juxtapositions of ice, land and ocean generate powerful evolutionary pressures and assembly filters that have created and sustained distinctive ecosystems.

Despite very limited diversity and restricted ranges of vascular plants and terrestrial vertebrates in Antarctica compared to elsewhere, terrestrial Antarctic ecosystems exhibit considerable cryptogamic, algal, invertebrate^5^ and microbial diversity^6^ across a range of physical environments and spatial scales. Over the past two decades, ecological, morphological and molecular studies^7,8^ have unveiled strong continental-scale biogeographic patterns^9^, evolutionary divergence partly reflecting glacial cycles and plate tectonics^10,11^, and high levels of species endemism^12,13^.

The continent is currently divided into 16 major Antarctic Conservation Biogeographic Regions (ACBRs^4,14^), roughly equivalent to global ecoregions^15^. These represent continental-scale patterns of biogeography, but each encompasses a mosaic of ecosystem types that reflect variations in biota and ecological processes along multiple environmental gradients expressed at a range of spatial scales. ACBRs alone, therefore, do not fully capture the diversity of ecosystems and functions that are represented across the continent, nor the functional and compositional relationships among different ecosystem types. For example, lowland systems adjacent to penguin and pinniped colonies become enriched in nutrients from marine subsidies^16^ affecting local biodiversity^17,18^, and this occurs all around the Antarctic coast through multiple ACBRs. Similarly, ice-free landscape features shaped by glaciers, such as moraines and nunataks, are widespread and may harbour functional similarities despite being spatially distant^19^. Finally, ACBRs do not explicitly represent less documented ecosystem components such as aquatic^20^ and microbial biota^6,21^.

In consequence, while the ACBRs form a valuable continental-scale foundation for assessing the conservation of Antarctica’s biodiversity^1,22–25^, their coarse resolution and inability to capture important ecological processes that shape the composition and functions of ecosystems limits their utility for finer scale conservation planning and management interventions. Hence, there is an urgent need to build on ACBRs to further develop a systematic environment-geographic framework for ecosystem management and area protection as envisaged by the Antarctic Treaty Consultative Parties in Annex V to the Environmental Protocol (https://www.ats.aq/e/protocol.html).

Two recent advances present a timely opportunity to initiate the development of such an inventory. First, comprehensive geospatial data sets for a range of environmental variables have become available and spatially explicit records of biota have been compiled and validated within a single archive^26^. This has enabled the development of habitat suitability models for key taxa across the continent^27^, providing the first consistent basis for biotic classification that represents significant variation and diversity of life that occurs at subregional resolution^12^. Second, the recently developed International Union for Conservation of Nature (IUCN) Global Ecosystem Typology^28^ established the conceptual basis for defining and classifying ecosystem types within a systematic hierarchy and for characterising the functional groups of ecosystems, their constituent biota and ecological processes. It establishes a global context for Antarctica, which includes the world’s greatest extent of ‘Ice sheets, glaciers and perennial snowfields’ (Ecosystem Functional Group T6.1) ‘Polar/alpine cliffs, screes, outcrops and lava flows’ (T6.2) and ‘Large seabird and pinniped colonies’ (MT2.2), as well as important southern outliers of ‘Polar tundra and deserts’ (T6.3) and ‘Freeze-thaw freshwater lakes’ (F2.4). All of these ecosystems are profoundly influenced by the Earth’s largest ice sheets, which influence climatic conditions, disturbance regimes and biogeographic insularity by forming a matrix around or adjacent to the ice-free ecosystems.

Our spatially explicit inventory of Antarctic ecosystems includes a hierarchical classification, systematic descriptions of the units, and rasters of their distributions throughout the continent. The classification comprises three tiers: broad environment types based on biophysical variables (Tier 1), biotic habitat types based on habitat suitability models for key biota (Tier 2), and bioregional expressions of habitat types based on ACBRs (Tier 3). Descriptions of habitat types were compiled from statistical summaries of environmental and biotic data, the literature, satellite imagery, and field-based expertise of the authors. Distribution maps were derived simultaneously with the classification.

The 3-tiered framework is designed to handle the limited quantity and biases of available biotic data and to facilitate updates with a modular workflow as knowledge advances. We expect environment types, which define the upper structure of the hierarchy, to be most robust to new information, since incremental improvements in environmental data layers are unlikely to alter understanding of the identity of major environments represented on the continent. We expect habitat types to be more sensitive to new information, including additional biotic records and alternative modelling methods, but quality assurance protocols employed in the model development should confer some robustness. For example, a rapidly expanding literature and accumulating samples of microbial biota across Antarctica may lead to refinements in this classification, although abiotic drivers in microbial distribution^6^ could also provide a source of validation. Lastly, we expect regional ecosystems to be most sensitive to new information, as improved field sampling supports or refutes hypothesised differences in biota between related ecosystem types delineated by current bioregional boundaries.

This new ecosystem inventory represents a transformational change in capacity to meet aspirations of the Environmental Protocol^2^. The data provide a stronger quantitative evidence base than has previously been available for spatial planning and analysis (e.g.^29^). Detailed analyses of the fine-resolution biodiversity information across the full range of ice-free environments in this inventory will support decision-making and risk assessment for environmental management, protected area design, infrastructure planning, environmental impact assessments, strategic threat abatement, and ecosystem management and restoration under the Protocol^30^. In particular, it will form a key input to systematic conservation planning analyses^31,32^ and inform the further development of the Antarctic Protected Area network. It will also enable more informed and efficient reporting of trends and status of Antarctic biodiversity, enabling the Antarctic Treaty system’s Committee for Environmental Protection to advise future Antarctic Treaty Consultative Meetings on the state of Antarctic ecosystems.

Our ecosystem typology and maps also provide foundations for a systematic risk assessment to identify Antarctica’s most threatened ecosystem types, diagnose the underlying causes of risk, and inform the design and implementation of policy and management strategies for risk reduction^33–35^. The resulting Red List of Antarctic ecosystems will be pivotal to monitoring and reporting on global biodiversity targets, will help to identify Key Biodiversity Areas and inform priorities for restoration of degraded ecosystems^36,37^.

More broadly, by characterising ecosystem types distinguished by fine-resolution variation in geophysical features and habitat suitability, the typology and maps will support the design and interpretation of ecological research by framing comparisons between like ecosystems across different regions and between contrasting ecosystem types within the same region. These datasets support the development and testing of generalisations about ecosystem responses to environmental gradients, management interventions and environmental change. Finally, placing Antarctic ecosystems in a global context through a systematic typology helps to communicate to a broader audience the major contribution that Antarctic protection can make to conserving the full variety of Earth’s ecosystems. This facilitates knowledge transfer among researchers and managers of like ecosystems in cryogenic environments and establishes a consistent vocabulary for education and knowledge transfer^28^.

## Methods

### Spatial extent

Ice-free areas were delineated by the union of the medium-resolution remote sensing-derived rock outcrop layer for Antarctica^38^ and the high-resolution Landsat-derived rock outcrop layer^3^. This ensured that as much ice-free ground as possible was within the scope of the ecosystem typology, as both layers have minor known omissions of ice-free areas due to limitations on methods or incomplete data. The union of these two layers was masked to land areas using a high-resolution Antarctic coastline polygon from the SCAR Antarctic Digital Database (https://add.scar.org/) to exclude areas where the mapped rock outcrop layers misalign with the coastline. This new ice-free layer is available in the Australian Antarctic Data Centre^39^. The total extent of the study area included 70,586 km^2^ of estimated ice-free ground. This is greater than the true ice-free area of Antarctica due to the resolution of the spatial data and the inclusion of all pixels overlapping the mapped ice-free area, including those with only small overlap at their edges.

### Data layers

Abiotic factors are predominant drivers of biotic distribution and abundance in Antarctica, with water availability, light, temperature, soil nutrients, disturbance regimes, and exposure to wind hypothesized as some of the key factors that shape Antarctic ecosystems^40–42^. The variables selected for ecosystem classification represented these ecosystem processes and had spatial data available at a continental scale. For example, insolation is a key determinant of energy supply for autotrophic organisms living in harsh environments and, together with wind exposure, also increases desiccation risk; length of summer growing season and meltwater supply limit the diversity and quantity of flora that can establish; and slope and precipitation influence the frequency and severity of avalanches. We compiled a set of ten environmental variables to represent the variation in physical components of ecosystems across Antarctica: elevation, slope, rugosity, wind, insolation, mean annual temperature, length of summer season, mean annual precipitation, melt and cloud cover (see Table 1 for data sources and details). The digital elevation model did not cover Elephant Island, Clarence Island, the South Orkney Islands, and the Balleny Islands, so these were excluded from the study. The South Orkney Islands are among the most biodiverse areas of the Antarctic and will likely require a separate analysis. A further four data layers included in Table 1 pertain to unique habitats that are not explicitly captured by other environmental data layers, including two for geothermally active areas (active volcanoes, currently dormant volcanoes and radiogenic sites), as well as penguin breeding areas and lakes.

**Table 1:** Spatial layers representing abiotic components of Antarctic ecosystems; includes ice-free areas used to define the typology extent, ten generic environmental variables and additional data on five Distinct Overlay environmental features.

Layer	Description/units	Primary data source	Citations	URLs
Ice-free rocks	Source data ice-free polygons were rasterized at 100 m resolution using ESRI ArcMap.	SCAR Antarctic Digital Database	Gerrish *et al*. 2020a	https://data.bas.ac.uk/items/077e1f04-7068-4327-a4f2-71d863f70064/
			Burton-Johnson *et al*.^3^	https://data.bas.ac.uk/metadata.php?id=GB/NERC/BAS/PDC/01394
			Tóth & Terauds^39^	https://data.aad.gov.au/metadata/AAS_4568_ice-free_rock_outcrop_union
Elevation	Raw elevation data in metres above sea level	Reference Elevation Model of Antarctica from University of Minnesota	Howat *et al*.^67^	https://www.pgc.umn.edu/data/rema/
				server: https://data.pgc.umn.edu/elev/dem/setsm/REMA/mosaic/v1.1/100m/
Slope	Elevation change in metres	Reference Elevation Model of Antarctica from University of Minnesota	Howat *et al*.^67^	https://github.com/AustralianAntarcticDivision/rema.proc
				server: http://data.raadsync.cloud.edu.au/rema_processed/100m_200m/
Rugosity	Terrain Ruggedness Index^79^	Reference Elevation Model of Antarctica from University of Minnesota	Howat *et al*.^67^	https://github.com/AustralianAntarcticDivision/rema.proc
				server: http://data.raadsync.cloud.edu.au/rema_processed/100m_200m/
Solar	Clear sky radiation corrected for the incident angle, diffuse and reflected radiation. Dependent on time of year and time of day, latitude, slope, and aspect. Relief shading is not considered. Average insolation in W/m2	Reference Elevation Model of Antarctica from University of Minnesota	Kumar *et al*.^68^	https://github.com/AustralianAntarcticDivision/rema.proc
				server: http://data.raadsync.cloud.edu.au/rema_processed/100m_200m/
Melt	CMS is the annual sum of the pixel days (units day km2) where melting occurs, times the pixel area (25 × 25 km2).” Layer is based on radiometric data collected 1979–2021, and has been interpolated to 1 × 1 km for this paper.	Cumulative melting surface (CMS) dataset by Picard and colleagues.	Picard *et al*.^70^; Torinesi *et al*.^71^	https://snow.univ-grenoble-alpes.fr/melting/
Mean Annual Temperature	Temperature at 2 m height from surface as average of 3-hourly model output over 2014 and 2015.	2014-2015 Antarctic Mesoscale Prediction System (AMPS) data	Powers *et al*.^69^	https://www.earthsystemgrid.org/dataset/ucar.mmm.amps.output.html
				https://www2.mmm.ucar.edu/rt/amps/information/archive_info.html
Summer Season (Degree days >−5 °C)	Number of degree days over −5 °C; derived from the mean annual temperature layer.	2014-2015 AMPS data	Powers *et al*.^69^	https://www.earthsystemgrid.org/dataset/ucar.mmm.amps.output.html
Total Annual Precipitation	Total snowfall per year in 2014-2015	2014-2015 AMPS data	Powers *et al*.^69^	https://www.earthsystemgrid.org/dataset/ucar.mmm.amps.output.html
Cloud	Fraction of sky covered by clouds as average of 3-hourly model output over 2014 and 2015	2014-2015 AMPS data	Powers *et al*.^69^	https://www.earthsystemgrid.org/dataset/ucar.mmm.amps.output.html
Wind	Wind speed at 10 m height from surface in m/s as average of 3-hourly model output from 2014 to 2015	2014-2015 AMPS data	Powers *et al*.^69^	https://www.earthsystemgrid.org/dataset/ucar.mmm.amps.output.html
Lakes	Polygon layer Antarctic lakes	British Antarctic Survey data catalogue	Gerrish *et al*. 2020b	https://data.bas.ac.uk/collections/e74543c0-4c4e-4b41-aa33-5bb2f67df389/
Rookeries	Point data for penguin rookeries, including species and records of penguin counts	Mapping Application for Penguin Populations and Projected Dynamics (MAPPPD)	open access database	https://www.penguinmap.com/
Non volcanically heated areas	There is only one area in Antarctica exhibiting radiogenic heat, the Broknes Peninsula in the Larsemann Hills. This site is represented by a single ice-free polygon in the rock layer.			
Volcanoes	Latitude/longitude data for confirmed volcanoes in Antarctica	Compiled from Global Volcanism Program,USGS Geographic names information system, and published books.	LeMasurier *et al*. 1990; Oliver *et al*.^73^; Boutron^74^; Palais *et al*.^75^; Esser & Kyle^76^; Lee *et al*.^77^	https://volcano.si.edu/database/search_volcano_results.cfm
				https://www.usgs.gov/us-board-on-geographic-names/antarctic-names

To represent biotic components of ecosystems, we obtained continent-wide habitat suitability layers for 25 taxa^27^ (Table 2). These layers were derived from area interaction models^43^, an extension of inhomogeneous Poisson point models^44,45^, and were built on over 30,000 continent-wide occurrence records collectively^26^. The use of spatial models to classify pixels, rather than developing a classification directly from biotic samples, reduces the influence of potential biases in the spatial distribution of biotic samples in this data-scarce region. Terauds *et al*.^27^ fitted models for 34 taxa initially. Groupings at various taxonomic levels were selected to balance functional differences between taxa with the need for sufficient records to statistically characterise the niche of the organisms, while avoiding conflation of taxa with markedly different niches. The model validation process using Monte Carlo based goodness of fit tests indicated good prediction power for 25 of the 34 models tested^27^, and these were included in our analysis. The nine excluded taxa (and others not considered due to lack of sufficient samples), while important components of Antarctic ecosystems, represented organisms whose occurrence could not be accurately predicted with the variables available. The included taxa were mainly rock and soil biota, as the focus here was on ice-free land, and aquatic systems were covered in a separate workflow (see Distinct Overlay Ecosystems).

**Table 2:** Taxa for which habitat suitability models were available^27^.

Common name	Incl	Phylum	Class	Order	Family	Genus species
Parisitiform mites	Yes	*Arthropoda*	*Arachnida*	*Mesostigmata*		
Acari mites	Yes	*Arthropoda*	*Arachnida*	*Sarcoptiformes*		
Acari mites	Yes	*Arthropoda*	*Arachnida*	*Trombidiformes*		
Springtails	Yes	*Arthropoda*	*Entognatha*	*Entomobryomorpha*		
Lichens	Yes	*Ascomycota*	*Lecanoromycetes*	*Acarosporales*	*Acarosporaceae*	
Lichens	Yes	*Ascomycota*	*Lecanoromycetes*	*Candelariales*	*Candelariaceae*	
Lichens	Yes	*Ascomycota*	*Lecanoromycetes*	*Lecanorales*	*Bacidiaceae*	
Lichens	Yes	*Ascomycota*	*Lecanoromycetes*	*Lecanorales*	*Cladoniaceae*	
Lichens	Yes	*Ascomycota*	*Lecanoromycetes*	*Lecanorales*	*Lecanoraceae*	
Lichens	Yes	*Ascomycota*	*Lecanoromycetes*	*Lecanorales*	*Parmeliaceae*	
Lichens	Yes	*Ascomycota*	*Lecanoromycetes*	*Teloschistales*	*Physciaceae*	
Lichens	Yes	*Ascomycota*	*Lecanoromycetes*	*Lecanorales*	*Stereocaulaceae*	
Lichens	Yes	*Ascomycota*	*Lecanoromycetes*	*Rhizocarpales*	*Rhizocarpaceae*	
Lichens	Yes	*Ascomycota*	*Lecanoromycetes*	*Teloschistales*	*Teloschistaceae*	
Mosses	Yes	*Bryophyta*	*Bryopsida*	*Bryales*		
Mosses	Yes	*Bryophyta*	*Bryopsida*	*Dicranales*		
Feather mosses	Yes	*Bryophyta*	*Bryopsida*	*Hypnales*		
Mosses	Yes	*Bryophyta*	*Bryopsida*	*Polytrichales*		
Mosses	Yes	*Bryophyta*	*Bryopsida*	*Pottiales*		
Green algae	Yes	*Chlorophyta*				
Chinstrap penguin	Yes	*Chordata*	*Aves*	*Sphenisciformes*	*Spheniscidae*	*Pygoscelis antarctica*
Gentoo penguin	Yes	*Chordata*	*Aves*	*Sphenisciformes*	*Spheniscidae*	*Pygoscelis papua*
Nematodes	Yes	*Nematoda*				
Diatoms and Xanthophytes	Yes	*Ochrophyta*				
Rotifers	Yes	*Rotifera*				
Adelie penguin	No	*Chordata*	*Aves*	*Sphenisciformes*	*Spheniscidae*	*Pygoscelis adeliae*
Petrels	No	*Chordata*	*Aves*	*Procellariiformes*		
Springtails	No	*Arthropoda*	*Entognatha*	*Poduromorpha*		
Mosses	No	*Bryophyta*	*Bryopsida*	*Grimmiales*		
Cyanobacteria	No	*Cyanobacteria*				
Tardigrades	No	*Tardigrada*				
Liverworts	No	*Marchantiophyta*				
Lichens	No	*Ascomycota*	*Lecanoromycetes*	*Lecideales*	*Lecideaceae*	
Lichens	No	*Ascomycota*	*Lecanoromycetes*	*Umbilicariales*	*Umbilicariaceae*	

The modelled groups were of different taxonomic levels based on functional uniqueness and data availability (e.g., penguins were modelled at species level, while arthropods were modelled at order level). Each row represents one habitat suitability model. The last nine models listed were removed by the screening process (see Methods text).

We transformed the habitat suitability layers to south polar stereographic projection and resampled to 100 m resolution grid using cubic interpolation in a geographic information system^46^. Interpolated layers were checked for outlier values that may result from the cubic interpolation process, but none were found.

### Tiered classification

**Fig. 1:**
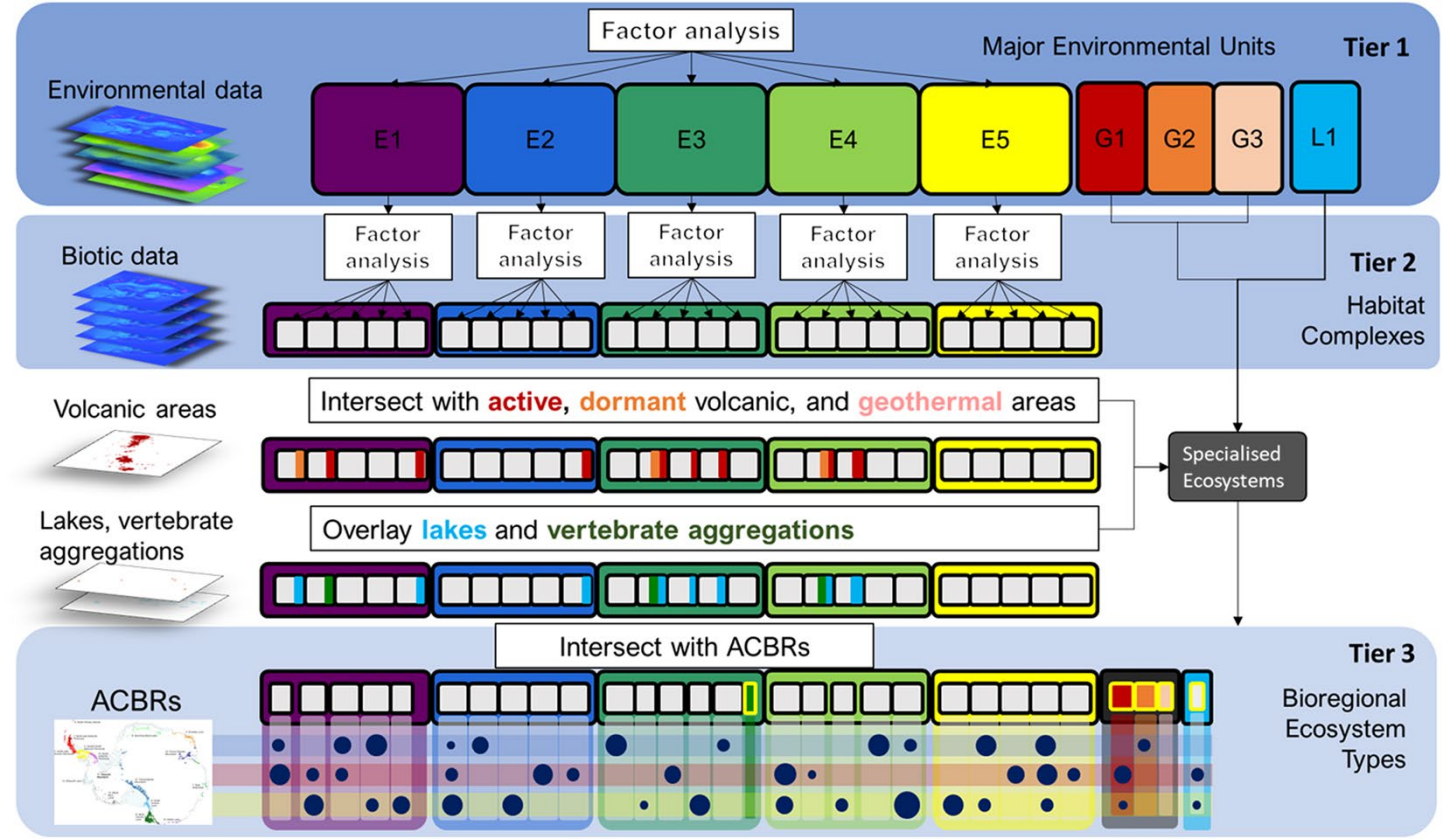
Graphic representation of the hierarchical classification process. Large, coloured cells (top row) represent Tier 1 Major Environmental Units (E1-E5); nested cells (middle rows) represent Tier 2, Habitat Complexes (including Distinct Overlay Ecosystems); and dark blue circles (bottom row) represent Tier 3, Bioregional Ecosystem Types. Geothermal Major Environmental Units are represented as red, orange, and pink nested cells. Penguin breeding areas are represented as a green cell nested within E1. The Lakes Major Environmental Unit (L) has one Habitat Complex (L1) represented by light blue cells. Antarctic Conservation Biogeographic Regions (ACBRs) in Tier 3 are illustrated by semi-transparent horizontal bands. Numbers and sizes of cells and circles are not to scale and are intended for illustration purposes only. Circles representing Bioregional Ecosystem Types have been placed to indicate that not all combinations exist, and existing combinations vary in size.

We developed a three-tiered analytical approach (Fig. 1) to capture continental-scale environmental patterns while also representing local-scale variations in habitats and ecosystem properties. Our approach was designed to develop an inventory of Antarctica’s ecosystems in the context of extreme limitations on data availability across an extensive region of remote, inhospitable and inaccessible territory. Ecosystem classification is therefore based primarily on environmental variables derived from models or satellite imagery, secondarily on habitat suitability models for key taxa and thirdly on bioregions as a proxy for spatial turnover in biodiversity.

The repeatable and readily updatable workflow presented here seeks to address a trade-off between the resolution of groupings and the robustness of those groupings to new information, given current constraints on data availability and reliability. Thus, tier 1 defines a small number of groups based on environmental landscape variables linked to ecosystem processes^47,48^ which, as methods of estimation improve, are unlikely to alter the major groupings. In contrast, tier 3 defines a finer thematic resolution with a large number of groupings, which are expected to be more sensitive to improvements in source data

The explicitly coded workflow linking the three tiers of classification (see Code Availability below) enables each tier to be updated independently in a transparent fashion when justified by improvements to particular components of the base data^47,49^. In contrast, traditional approaches to ecological classification and mapping (e.g.^50^) rely more heavily on subjective delineation by experts, for which updates are difficult to undertake and document in a transparent manner.

### Factor analysis

First, all pixels containing ice-free areas were classified using a factor analysis based on the 10 environmental (abiotic) variables listed in Table 1. We defined the resulting groups as ‘Major Environmental Units’ comprising Tier 1 of the typology. Second, each Major Environmental Unit was sub-divided based on a separate factor analysis of the 25 habitat suitability maps for biota. The resulting subgroups defined as ‘Habitat Complexes’ comprise the first part of Tier 2 of the typology.

The factor analysis used in Tiers 1 and 2 is a method of gradient partitioning similar to principal component analysis but allowing for unique variance in the input variables^51,52^. Factor analysis fits latent variables to high-dimensional input data and produces loadings that represent the relationships between the measured variables and the latent variables. The latent variables reflect recurring combinations of input variables, or environmental ‘facets’ of the data, and the loadings are used to calculate scores describing the alignment of each sample (in our case, each pixel) with these facets. We produced a deterministic cluster solution by classifying pixels according to the latent variable with the highest score. This approach ensures that each pixel is classified according to the latent variable or facet which affects it the most strongly. For example, if the first latent variable represents a moisture gradient (as was the case here), it ensures that all pixels that are characterised most strongly by moisture availability (for example, high precipitation, melt, and/or cloud cover) are placed in the same group. More arid pixels are classified according to variables that affect them more strongly than moisture (for example, relatively mild temperatures, high terrain variability, or high sunlight availability).

High negative scores for the facets are customarily classified into separate groupings, but we did not follow this convention for several reasons. First, high positive values for four out of five facets corresponded to conditions that promote diversity (e.g. higher moisture availability, lower elevation/warmer temperatures, terrain heterogeneity, or greater sunlight availability). Second, scores for three out of five facets were right skewed in their distributions, suggesting that separating the high tails groups is ecologically relevant and produces groups that are separated more clearly. Though the facets were uncorrelated, their underlying drivers yielded combinations of environmental conditions that made classifying by high values more practical. For example, terrain ruggedness becomes more variable at higher elevations, making it more practical to classify low elevations into one group, but separate high elevation areas by ruggedness. For habitat suitability facets, the highest scores were once again used because all loadings were positive and we wished to classify Habitat Complexes based on predicted presence, not absence, of taxa.

We used a combination of existing approaches to determine the number of factors used to partition groupings in Tiers 1 and 2, including an evaluation of matrix eigenvalues and parallel analysis^53,54^. The latter is a null-model approach which compares the eigenvalues of the data to those of a series of random datasets with the same attributes.

**Fig. 2:**
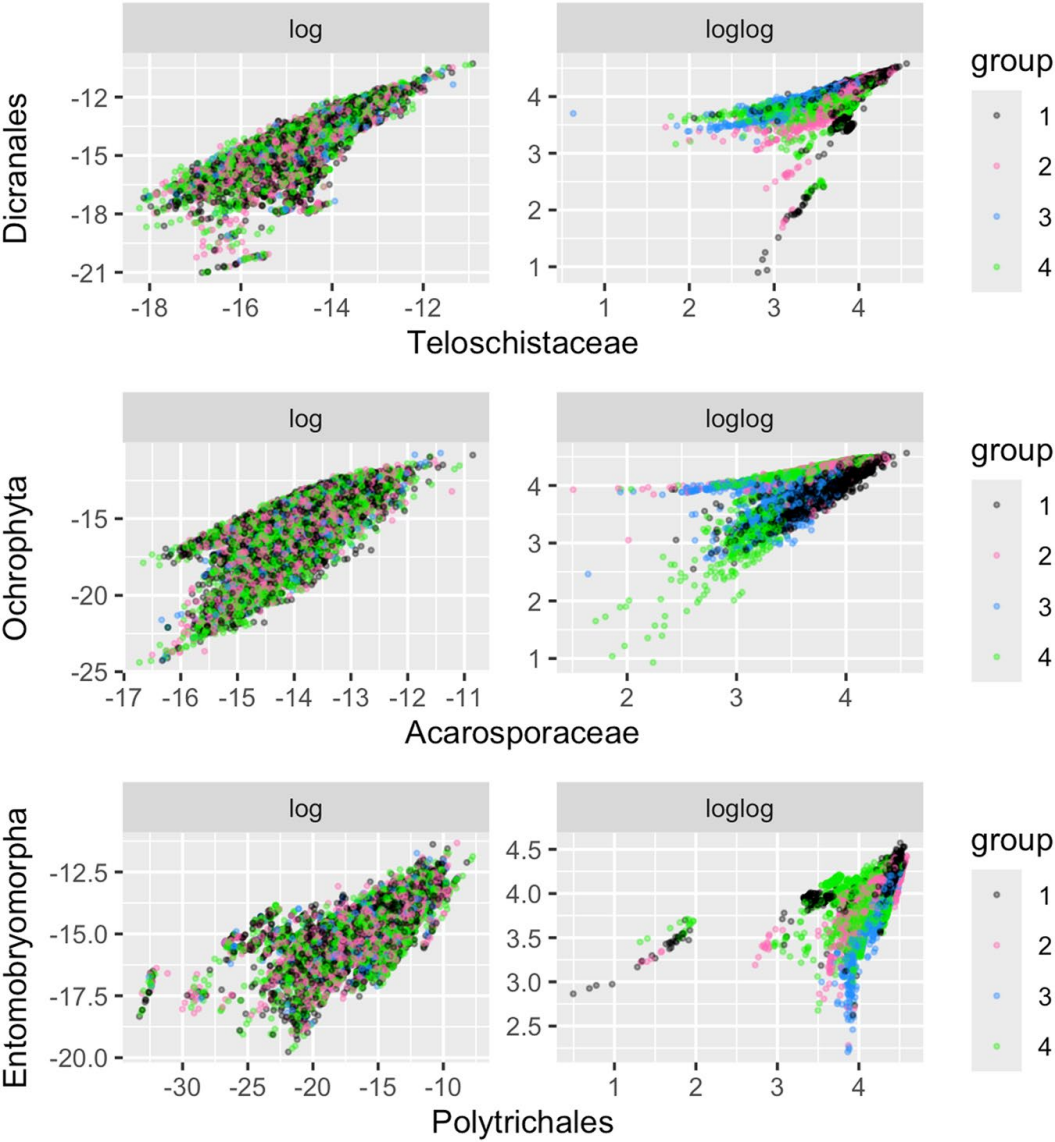
Tier 2 data transformations. Segregation of habitat groupings within Major Environmental Unit E2 by factor analysis classification following the same procedure on single-log (left) and double-log (right) transformation of predicted abundance from habitat suitability models. Plots were based on a random sample of 20,000 pixels for visualisation. The colours represent the four habitat complexes into which E2 pixels were classified. The x and y axes represent the habitat suitability values for six randomly chosen taxa. Results for all Major Environmental Units and any combination of taxa reflect similar differences between the coherence of the classification between the single- and double-logged data.

The second factor analysis to define Tier 2 groupings was based on predicted abundance from habitat suitability models listed in Table 2^27^. Due to low diversity and biomass in many areas of Antarctica, these data were highly skewed toward very small values and were furthermore autocorrelated due to the harsh environmental gradients (i.e. most organisms become less abundant when it is colder, dryer, etc.). We therefore implemented a series of transformations to accentuate the differences in the distributions of habitat suitability values. A single log transformation was insufficient to produce ecologically interpretable separation of groupings based on biotic composition (Fig. 2 – left side). We therefore performed a range normalisation of values to fall between 1 and 100 and a second log transformation, which emphasized the differences in species abundance on a relative scale, producing ecologically interpretable groupings (Fig. 2 – right side). The wedge shape with the point in the top right of all plots in Fig. 2 indicates nestedness – virtually all taxa have their greatest habitat suitability in the mildest conditions. Beyond these conditions (i.e. to the left of the top-right point) the ‘cross-correlations between taxa break down and, for any given density of records for one taxon, the other may be recorded at a range of densities.

A small number of pixels (<3%) had environmental data but lacked habitat suitability data due to coarse resolution at the margins of our study area. Thus, they were placed in Tier 1 groups by the first factor analysis but could not be classified in the second factor analysis. These unclassified pixels were assigned to the same Habitat Complex as their nearest neighbouring classified pixels in the same Major Environmental Unit using the ArcMap function Nibble. Isolated unclassified pixels that were not in a contiguous patch of ice-free land with a classified pixel were left unclassified (~1.4% of pixels).

Our methods impart several advantages over the alternatives that we considered. First, factor analysis is deterministic and repeatable. It does not rely on randomizations as do many more sophisticated algorithms, which can produce very different results if run multiples times over datasets with no clear transitions, as is the case here. Second, factor analysis is highly interpretable. By examining the loadings, we can easily determine the primary features of each group (Tier 1 loadings are presented in Table 3 and Tier 2 loadings are presented in Table S1). Third, the approach is flexible, for example by allowing non-orthogonal latent variables unlike many other multivariate analyses. Fourth, we deal with inherent limitations and biases in the data, for example, the use of habitat suitability models reduces the effects of spatial biases in biodiversity sampling. Fifth, we capitalize on the characteristics and features of the dataset by incorporating the often high covariances of our input variables (e.g. elevation correlates with temperature) while highlighting any differences between them. Finally, the tiered approach aligns with the Global Ecosystem Typology and provides a context for functional similarities and differences between ecosystems, rather than a simple list of types.

**Table 3:** Loadings for Tier 1 of the factor analysis representing the importance of each abiotic variable for scoring the fit of a pixel into each Major Environmental Unit.

variable	E1	E2	E3	E4	E5
cloud	0.121	**0.775**	0.017	0.065	−0.110
wind	−0.045	−0.046	0.127	−0.161	**0.469**
meanTemp	**0.489**	**0.671**	0.194	0.071	−0.241
melt	0.268	**0.502**	0.129	0.040	−0.225
elevation	−**0.959**	−0.228	−0.078	0.138	0.042
rugosity	−0.038	0.120	−0.039	**0.955**	−0.121
slope	−0.045	0.082	−0.031	**0.965**	−0.158
totPrecip	0.071	**0.947**	0.101	0.140	0.253
solar	0.112	0.147	**0.697**	−0.051	0.149
DDm5	**0.549**	**0.574**	0.171	0.067	−0.036

Scores were calculated for E1-E5 for each pixel, and the highest score, representing the best fit, determined the classification of the pixel. Loadings greater than 0.45 are bolded to emphasize the main features of each Tier 1 unit.

### Distinct overlay ecosystems

Some distinct ecosystem types described in the literature could not be reliably identified and mapped by the factor analysis of the general environmental and biotic spatial data. We used other data sources to map some of these ecosystems (Table 1), including lakes, bird and seal breeding habitat, and geothermal areas. These were added to the typology, described and mapped using separate workflows (Fig. 1).

#### Geothermally active areas

Geothermally active areas have distinctive natural disturbance regimes, thermal and geochemical properties which support unique microbiota, flora and fauna and maintain ice-free refuges. A point dataset of known volcanoes (including inactive and dormant volcanoes), geothermal areas and information on their history was compiled from internet and literature sources^55^ (Table 1). Each geothermally active area was manually circumscribed by a polygon from imagery available in Google Earth Pro. These manually delineated polygons were then clipped to ice-free land areas for inclusion in the map. Using information on volcano history, we classified all geothermal areas into the following four categories:

Active - volcanoes that have erupted in the past 1000 years, including areas that have been observed with fumaroles, or that likely serve as a source of geothermal heat. Mt Terror and Mt Terra Nova were included in this group despite older activity due to their close proximity to Mt Erebus, whose eruptions have likely affected them.

Dormant - Older volcanoes that last erupted 1000–100,000 years ago and are likely to have enriched volcanic soils.

Inactive - Volcanoes for which the youngest dated rocks/eruptions are older than 100,000 years, or their eruption history is unknown. These were excluded from the geothermal ‘Distinct Overlay Ecosystems’ Major Environmental Unit, allowing their classification and mapping to be determined by the factor analyses described above.

Radiogenic - A single example is currently known in Antarctica, the Broknes Peninsula in the Larsemann Hills, which exhibits radiogenic decay of Cambrian rocks^56–58^.

Active, dormant, and radiogenic sites were each included in the typology as a Major Environmental Unit representing geothermal ecosystems (G1-3; Fig. 1). Inactive volcanoes were excluded and instead assigned to various Habitat Complexes as determined by the factor analysis.

#### Seabird and seal breeding areas

Aggregations of marine vertebrates, including penguins, other seabirds and pinnipeds, are characterised by very large influxes of marine nutrients and local habitat modification. Emperor penguins and most seal species breed on ice with very few exceptions (one known Emperor penguin colony at Taylor Glacier in the Prince Charles Mountains) and are therefore not components of ice-free ecosystems. Among pinnipeds, elephant seals are the only group to regularly use terrestrial sites for moulting and breeding. Therefore, a complete mapping of ice-free breeding areas will include colonies of gentoo, chinstrap, and Adèlie penguins, migratory seabirds, as well as elephant seals. Relatively comprehensive data were available for breeding colonies of penguins as point data (837 colonies from penguinmap.com). Data for other vertebrate breeding areas (pinnipeds and other seabird colonies) are currently limited, but these areas will form part of the Habitat Complex as more data become available. The land-breeding penguin data were cleaned to (i) remove records that were farther than 4 km from the nearest mapped land (one colony), (ii) manually adjust co-ordinates of records that were 3–4 km from land (two colonies), and (iii) adjust to the nearest coastline records that were less than 3 km from the coast (236 colonies, including five that were more than 1 km from a coast). Large seabird colonies (80,000 + breeding pairs, n = 14) whose positions were adjusted were then checked against satellite imagery in Google Earth Pro to manually ensure accuracy of the resulting dataset. Seabird colonies smaller than this threshold were not consistently distinguishable from surrounding rock from satellite imagery via guano stains.

PenguinMap includes estimates of population size for each colony based on nest count, adult count, and/or chick count. These estimates could be derived from data collected over multiple years. To estimate the area covered by each colony, we averaged each type of data for each colony over time and standardised between the different count types. We calculated the mean ratio of chicks:nests and adults:nests for each species where observations were taken from the same colony in the same year. We then repeated the process using averages over all observed years for a given colony; this produced similar values for those that could be cross validated against the yearly resolution method and provided estimates for adult:nest ratios for chinstrap penguins, which could not be calculated at the yearly resolution. To calculate a final estimate for breeding pairs per colony, we equated nest count with breeding pairs, then used the adult/chick counts divided by the ratios to estimate breeding pairs for sites where there was no nest count available.

Colony population size thus standardized, we translated each colony’s population to a spatial footprint estimate (area in hectares, since each grid square was one hectare). For this we used a dataset of penguin colony sizes and spatial extent (Table 1 in^59^). Colony population size has previously been shown to explain over 98% of variation in colony spatial extent^60^ in a linear relationship, so we fitted a linear regression of breeding pairs to colony size and used the regression model to calculate colony size, *a*, in square metres from the estimated number of breeding pairs. For each colony, we used the estimated area to calculate a buffer radius, $r=2\sqrt{a/\pi }$, which includes a multiplier of two because most colonies are in direct proximity to the coastline, and these lose about half of their area assuming a circular buffer over a straight coastline. Furthermore, for the purposes of ecosystem mapping, it is likely that nutrients built up over years (sometimes decades or centuries) of use by penguins will influence an area larger than the area directly occupied by nests, and therefore warrants an expanded area of influence of up to hundreds of metres from the colony boundaries^17^. The colony point data were buffered by *r* and clipped to land areas. Colonies larger than 80,000 breeding pairs (n = 14) were once more checked and adjusted manually, if required, to correspond with published maps^61^ or to encompass guano stains visible from satellite imagery (three colonies adjusted). The final polygons representing the spatial extent of Antarctic penguin colonies were converted to raster format such that any pixel intersecting with a colony was classified as a colony.

#### Lakes

A lake layer with high-resolution polygons was downloaded from “a very incomplete dataset of surface lakes in Antarctica” the SCAR Antarctic Digital Database^62^ and masked to land surfaces. This layer was converted to a binary 100 m raster in which any pixel containing 50% or more coverage by a lake (Table 1) was classified as a lake. The minimum size for a lake to appear in this raster was a circular lake 80 m x 80m in diameter, corresponding to an area of ~5,000 m^2^, or half a pixel (100 × 100 m = 10,000 m^2^).

According to median lake sizes^63^ in the McMurdo Dry Valleys Antarctic Specially Managed Area (ASMA), this essentially excludes kettle lakes from representation in the raster (these were not in the polygon layer to begin with), which comprise the vast majority of all lakes in the ASMA (93%). They are linked with areas of recent glacial activity such as glacial outwash plains and moraines. Fortunately, these glacial features are also individual Habitat Complexes in Tier 2 of the typology. Because kettle lakes appear to be a major feature of glacially-formed Habitat Complexes and are also ephemeral on decadal to centuries time scales^63^, future studies will have the option of incorporating kettle lakes as a feature of glacially formed Habitat Complexes, or as a distinct ecosystem.

Topographic lakes in the ASMA, vary greatly in size, origin and description. Lakes frozen to the base (n = 11) tend to be larger, so were mostly included. Of the remaining topographic lakes, glacial lakes tended to be larger than non-glacial lakes, and more than half of the latter were larger than 0.5 ha. Hundreds of lakes from East Antarctica, including the Vestfold Hills, were detectable at the 100 m resolution, and these vary broadly from hypersaline lakes that are ice-free year-round to freshwater oligotrophic lakes. Besides excluding smaller lakes, the 100 m resolution also implies that the shapes and sizes of these lakes will not be exact the raster layers. It should be emphasized that lakes, streams, and other aquatic habitats are an important, distinct feature of any comprehensive typology, regardless of the complexities of mapping and resolution encountered when embedding them into a spatial representation of the landscape. They provide habitat for diverse microbiota and specialised chemotrophic processes in areas where few organisms can survive terrestrially. The way lakes were included in the 100m raster means that shoreline areas, which are hotspots of microbial diversity often embedded in barren deserts, are not consistently classified. These areas should be mapped at finer resolutions for the purposes of any future assessments and their role connecting aquatic and terrestrial habitats carefully considered.

### Assembling Tier 2 spatial data

The output of the biotic factor analyses formed the base layer from which the ecosystem map of ice-free areas was assembled, with each cell attributed with Habitat Complex and Major Environmental Unit. Sequentially, the geothermal layer was overlaid on the factor analysis output, followed by the penguin colony raster and the lakes raster. The mapped Habitat Complex was determined by the uppermost layer. Single Habitat Complexes were recognised in each of the four Major Environmental Units for Distinct Overlay (geothermal and lake) Ecosystems (Fig. 1). Bird and seal habitats (based on available data for penguin breeding colonies on ice-free ground) were included in the typology as a Habitat Complex within Major Environmental Unit E1, which has the lowest elevation.

### Bioregional ecosystem types (Tier 3)

**Fig. 3:**
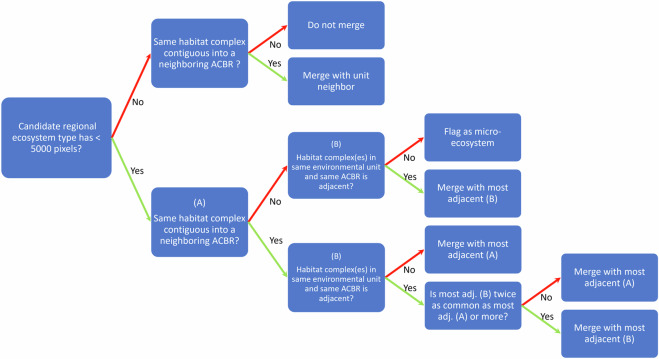
Amalgamation rules for Bioregional Ecosystem Types. Decision tree for lumping candidate bioregional combinations of Habitat Complex and ACBR into the final Bioregional Ecosystem Types. At least 5% of pixels in the candidate unit in question need to be next to a pixel of another unit to be considered “adjacent”. Red arrows are followed upward if the condition is not met, and green arrows are followed downward if it is.

We expected strong regional turnover in the biotic composition (beta and gamma diversity) within Habitat Complexes, especially given the insularity of many ice-free areas, intrinsic limitations on dispersal of many organisms and habitual use of breeding sites by most vertebrates. To represent this variation in Tier 3 of the typology, we used ACBRs as simple proxies for regional-scale biotic turnover within Habitat Complexes to develop a finer level of classification (Bioregional Ecosystem Types). We first performed a spatial intersection of Habitat Complexes with ACBRs. This resulted in 398 candidate units, some with as few as a single pixel. We reviewed these candidate ACBR-habitat combinations using decision rules (Fig. 3) to identify and merge marginal units that may be artefactual fragments of the intersection process. Thus, ACBR-habitat combinations with small areas were merged with adjacent units in the same Major Environmental Unit, resulting in a total of 267 Bioregional Ecosystem Types across ice-free Antarctica, including 29 with Distinct Overlay Ecosystems.

## Data Records

### Access

The Antarctic Ecosystem Inventory v1.0, including spatial data^64^ and descriptions^65^ for Major Environmental Units and Habitat Complexes, is available via the Australian Antarctic Data Centre (https://data.aad.gov.au/metadata/AAS_4568_The_Antarctic_Ecosystem_Inventory) and on Zenodo (10.5281/zenodo.11629115, 10.5281/zenodo.12737213). Data are released under a Creative Commons Attribution 4.0 International licence enabling reuse with attribution via a citation of this descriptor. We expect the inventory to be an evolving data product, with successive versions released in future as knowledge develops and new data become available. Labelling uses semantic versioning to indicate the change between versions.

Spatial data for input variables used in the analyses are listed in Table 1 and are available from the following sources: https://www.arcgis.com/home/item.html?id=f030d958beb6482f9fd1bb47847ac3f9 for the Antarctic coastline^66^; https://data.aad.gov.au/metadata/AAS_4296_Antarctic_Conservation_Biogeographic_Regions_v2 for ACBRs^4^; https://data.aad.gov.au/metadata/AAS_4568_ice-free_rock_outcrop_union for ice-free areas^39^; https://data.aad.gov.au/metadata/AAS_4568_The_Antarctic_Ecosystem_Inventory with sub-directory “Abiotic Variables” for all abiotic variables including terrain, processed climate data, and melt data, the original versions of which can be accessed online at https://github.com/AustralianAntarcticDivision/rema.proc for terrain data^67,68^, https://www.earthsystemgrid.org/dataset/ucar.mmm.amps.wrf_30.html for climate data^69^, and https://snow.univ-grenoble-alpes.fr/melting/ for melt data^70,71^; https://data.aad.gov.au/metadata/AAS_4296_Antarctic_terrestrial_biodiversity_DB for the full biodiversity occurrence records^26^ and https://www.penguinmap.com/ for penguin colony data; https://data.bas.ac.uk/collections/e74543c0-4c4e-4b41-aa33-5bb2f67df389/ for the lakes layer^62^; https://data.aad.gov.au/metadata/AAS_4296_Environmental_drivers_of_Antarctic_biodiversity_in_ice-free_areas for interpolated habitat suitability layers^27^. Datasets generated during this research are included in the GitHub repository (volcano^72–77^ data and edited penguin colony data) at https://github.com/anikobtoth/Antarctica.

### Format and nomenclature

Definitions of major terms in the datasets are as follows:

**Antarctic Conservation Biogeographic Regions (ACBRs)** – the broad scale, ice-free regions of Antarctica with biogeographically distinctive biota. These were defined by Terauds *et al*.^14^ and Terauds and Lee^4^ using a combination of expert opinion and analysis of selected biological groups well-represented in the biodiversity of ice-free Antarctica database (Terauds *et al*.^26^).

**Major Environmental Units** – the top tier of the ice-free ecosystem typology representing environments that share major climatic and geomorphological features across the continent.

**Habitat Complexes** – the second tier of the ice-free ecosystem typology representing local variations in combined habitat suitability for a range of vertebrates, invertebrates, plants and lichens. Habitat Complexes are nested within Major Environmental Units. They reflect expected similarities in the structure of biotic communities over local scales and may extend across multiple ACBRs.

**Bioregional Ecosystem Types** – the third tier of ice-free ecosystem typology representing regional expressions of Habitat Complexes derived from their intersection with ACBRs, which are assumed to represent regional patterns in biotic composition. These types are consistent with Level 4 units in the IUCN Global Ecosystem Typology^28^. They serve as proxies representing the variation in ecosystem processes and compositions across the continent.

**Distinct Overlay Ecosystems** – ecosystem types associated with distinct environments that are not adequately represented in environmental data layers used to define other ecosystem types in the typology.

Units in all three tiers of the typology were labelled with semantic codes. Membership in Major Environmental Units of Tier 1 is designated by ‘E’ for ‘environment’, ‘G’ for geothermal, and ‘L’ for lakes. Membership of Habitat Complexes in Tier 2 is designated by ‘B’ for biotic. In Tier 3 ‘Bioregional Ecosystem Types’ were labelled with their Habitat Complex code string combined with their respective ACBR abbreviation (Table 4). Thus, for example, E2B1-NWAP denotes Midland mesic environments (E2) with Habitat Complex B1 in the Northwest Antarctic Peninsula ACBR. Where fragments of one or more Habitat-ACBR combinations were rationalised with an adjacent, more extensive combination, the code for the more extensive combination was adopted as the label for the resulting Bioregional Ecosystem Type and appended with ‘a’ to indicate amalgamation.

**Table 4:** Antarctic Conservation Biogeographic Regions (ACBRs) v2.0^4^ with abbreviations used in codes for Bioregional Ecosystem Types.

Antarctic Conservation Biogeographic Region	Abbreviation
1 North-east Antarctic Peninsula	NEAP
2 North-west Antarctic Peninsula	NWAP
3 Central south Antarctic Peninsula	CSAP
4 Enderby Land	EDL
5 Dronning Maud Land	DML
6 East Antarctica	EA
7 North Victoria Land	NVL
8 South Victoria Land	SVL
9 Transantarctic Mountains	TM
10 Ellsworth Mountains	EM
11 Marie Byrd Land	MBL
12 Adélie Land	AL
13 Ellsworth Land	EWL
14 South Antarctic Peninsula	SAP
15 Prince Charles Mountains	PCM

The descriptions of nine Major Environmental Units in Tier 1 include distribution maps showing their component Habitat Complexes and statistical summaries (as box plots) of all environmental and biotic variables used in the factor analyses^65^. Descriptive profiles prepared for each of 33 Habitat Complexes include general text commentaries, photographic images, text description and local maps of distribution, text descriptions and statistical summaries of their environmental properties, and tabulated summaries of biotic records within the mapped area using the biodiversity occurrence database, as well as contextual data in relation to other ice-free areas of Antarctica and areas within the same Major Environmental Unit. Tier1 and Tier 2 descriptions are also compiled in Table S2. Descriptions of Biogeographic Ecosystem Types (Tier 3) comprise a written summary and figures depicting distribution among ACBRs. The 269 Biogeographic Ecosystem Types (Tier 3) were rationalised from a total of 369 candidate factorial combinations of Habitat Complex and ACBRs (see Methods; Fig. 3). They align with Level 4 of the IUCN Global Ecosystem Typology^28^.

The spatial data for all three tiers of the ecosystem typology are supplied as a 100 m raster in the South Polar Stereographic projection. The raster is accompanied by a variable attribute table, enabling display, query and analysis of units within each of the three tiers of classification. Raster values are also provided in Table S2.

### Coverage

**Fig. 4:**
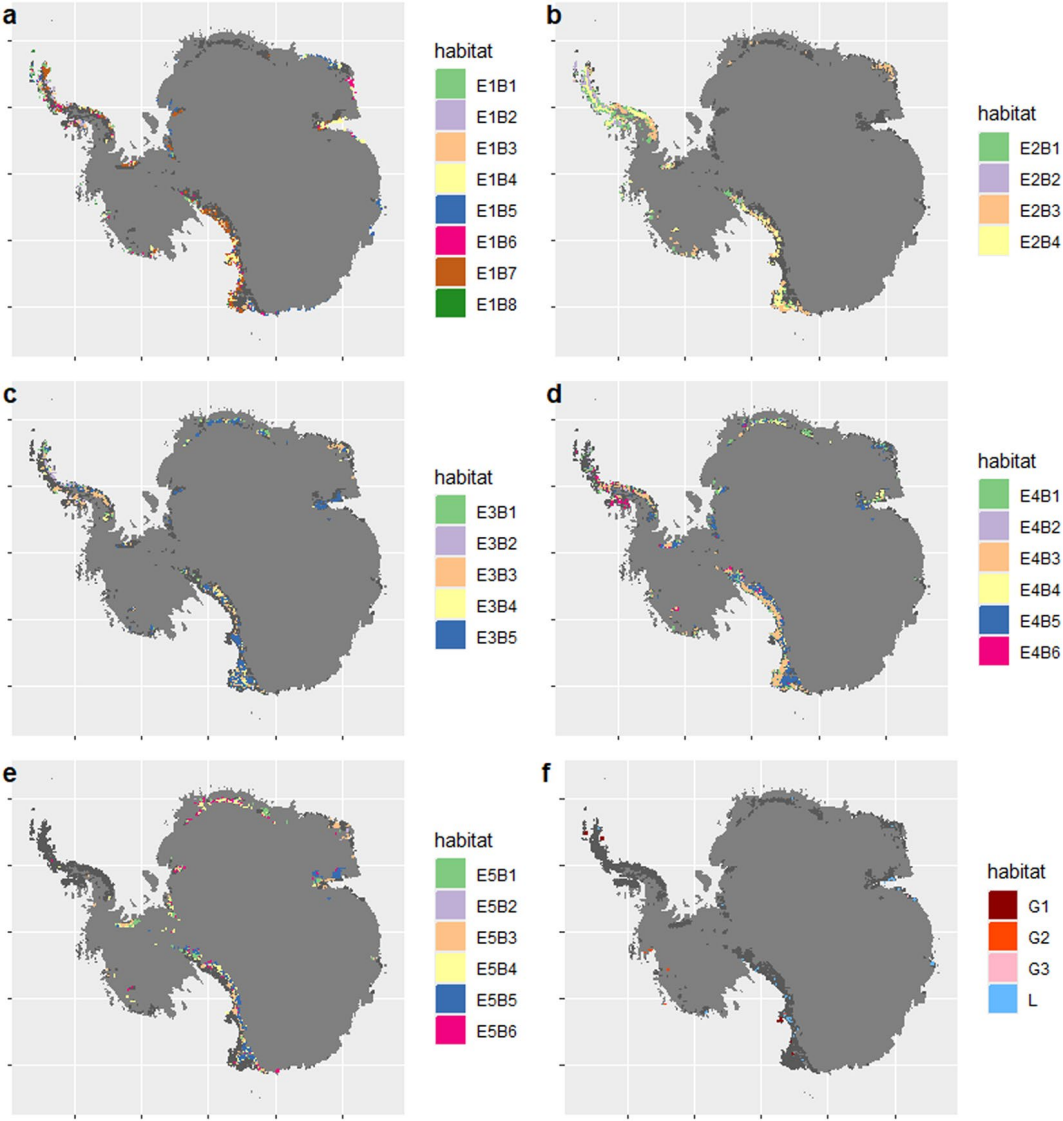
Spatial distribution of Habitat Complexes within each Major Environmental Unit (top left to bottom right): E1 mild lowlands; E2 midland mesic environments; E3 midland dry sunny environments; E4 rugged high mountains; E5 highland windy plateaus; and G1-G3 geothermal ecosystems with L1 lake ecosystems. Dark grey pixels on each map indicate distribution of ice-free areas assigned to other Major Environmental Units.

**Fig. 5:**
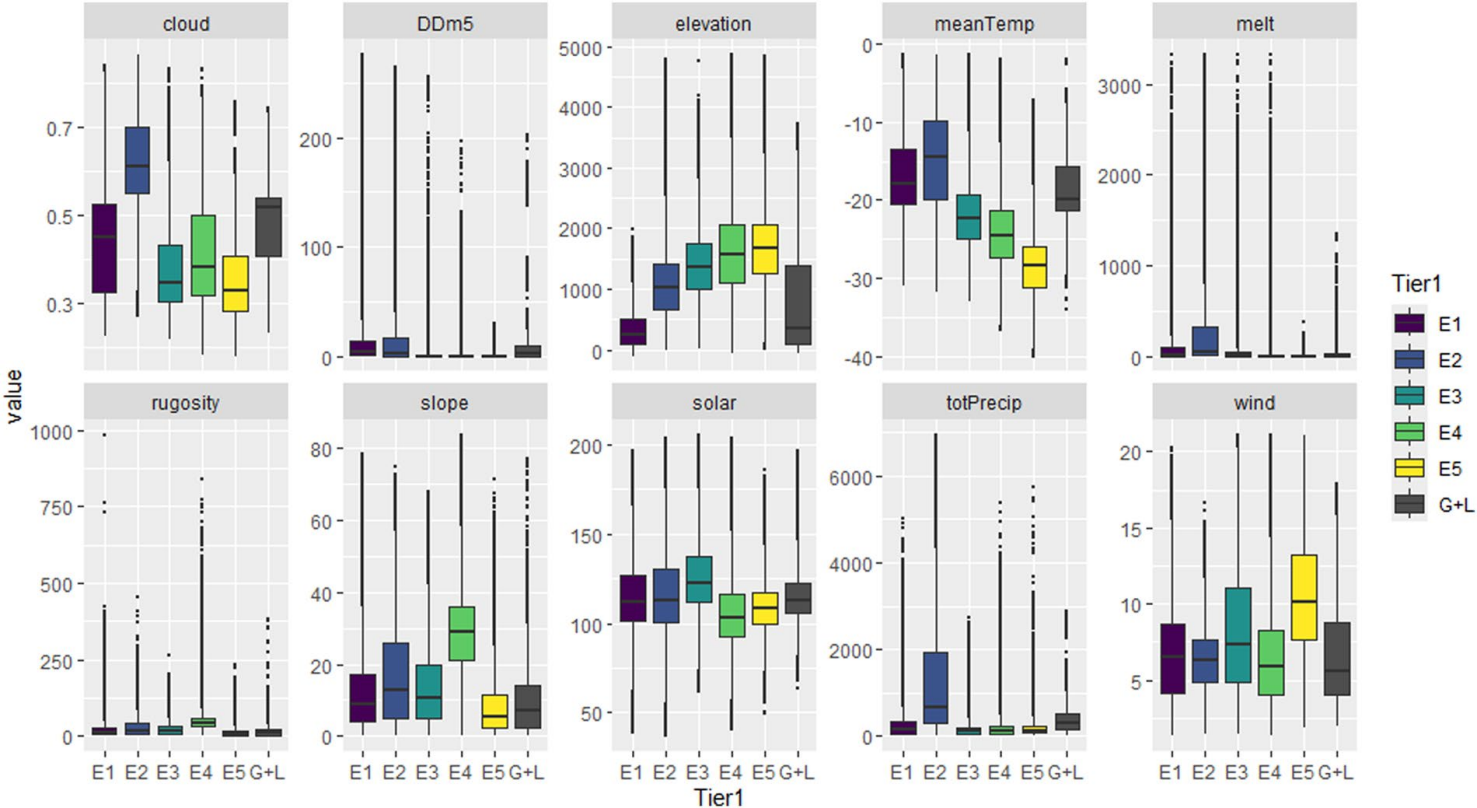
Environmental properties of Major Environmental Units. Cloud, melt, and totPrecip are moisture availability variables. MeanTemp is the mean Temperature during 2014–2015 and DDm5 is the degree-days over −5 °C, a common measure of the growing season. Solar is solar radiation calculated from aspect, reflected light, and other factors. Elevation, rugosity and slope are terrain variables based on the digital elevation model (Table 1); and wind is an average measure of wind speed.

**Fig. 6:**
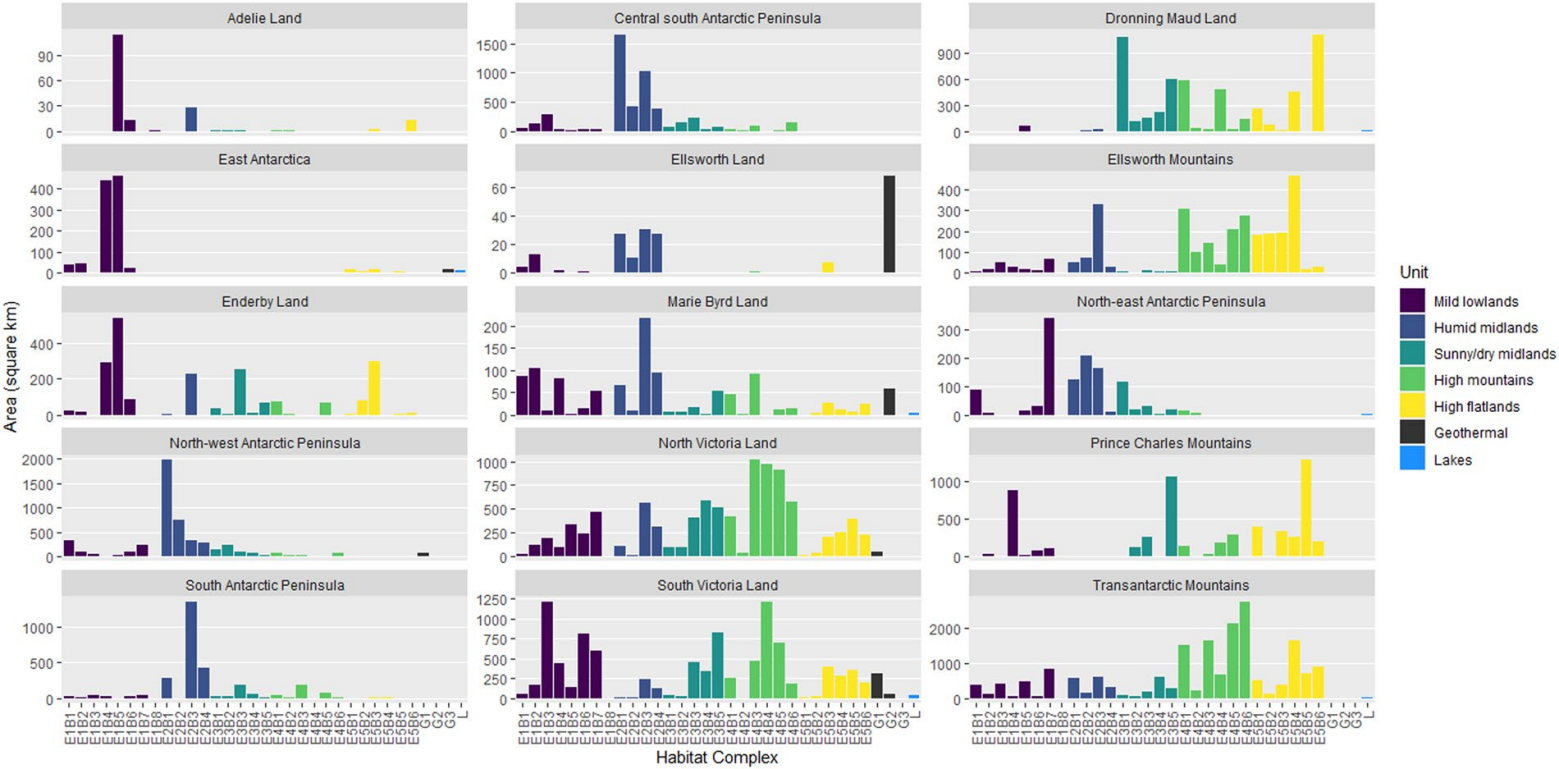
Regional representation of Habitat Complexes among Antarctic Conservation Biogeographic Regions (ACBRs) coloured by Major Environmental Unit. Note that the South Orkney Islands ACBR is not included.

The data cover ice-free areas of terrestrial Antarctica, as circumscribed in Methods (Spatial extent) and shown in Fig. 4. A summary of the environmental conditions that comprise each Major Environmental Unit (Tier 1) is shown in Fig. 5, and the distribution of Bioregional Ecosystem Types (Tier 3) is summarised in Fig. 6.

## Technical Validation

Input layers underwent independent validation procedures detailed in their respective source publications (Table 1). The spatial distribution of ice-free areas was initially based on mapping by Burton-Johnson *et al*.^3^, who estimated mean overall accuracy of 85% (±SD 8) based on manually delineated exposed rock areas. Our inclusion of additional ice-free areas mapped by Gerrish *et al*.^38^ is likely to have reduced errors of omission. Details of validation procedures for other environmental data layers used as inputs into factor analyses are available from their published sources (Table 1).

Habitat suitability models were based on occurrence records that conform with data standards applied in the Biodiversity of Ice-free Antarctica Database. These data are subject to inevitable biases in sampling coverage due to access constraints in very remote areas. The models for 34 taxonomic groups were screened using Monte Carlo based goodness of fit tests procedures comparing the original occurrence data to the spatial realisation of each model^27^. Screening identified models for 25 of the 34 taxa that performed sufficiently well in this comparison for use in the factor analysis (Table 2).

Our three-tiered analytical framework enabled structured checking and evaluation of successive classification and mapping outputs. These were reviewed by Antarctic experts on the authorship team, initially at a face-to-face workshop, and subsequently by correspondence, with iterative adjustments to resolve identified anomalies. We produced a series of visualisations, statistical summaries and maps of the properties for all three tiers of classification units^65^ as a basis for critical review by the author team. This review process resulted in iterations of the factor analysis to optimise the set of input data layers, for example, by exploring alternative transformations of biotic data to optimise the resolution of Habitat Complexes (Fig. 2). We also merged an early draft ‘coastal environmental unit’ which was poorly discriminated from the adjacent lowland unit. Expert appraisal of the outputs also identified the need to address Distinct Overlay Ecosystem types (geothermal systems, vertebrate breeding areas and lakes) in separate workflows to ensure accurate representation.

Our approach to landscape classification incorporated geophysical, biotic, and biogeographic variation in an explicit workflow under severe data availability constraints. We were able to supplement the typology with data on special features, such as seabird breeding areas, geothermal areas, and lakes, which were not well-represented in the environmental data set or habitat suitability models. The use of habitat suitability models, screened for reliability, enabled us to exploit a growing biotic inventory for the Antarctic (e.g.^78^), while reducing spatial biases stemming from logistic and economic constraints on field access that precluded continent-wide classification of the raw data.

Our data product could not undergo a traditional validation process, due to the extreme logistical constraints on access to most of Antarctica and the resulting scarcity of suitable ecosystem observations for validation. Instead, experienced field scientists on the author team reviewed the mapping output and we calculated a confidence index based on the dominance of the best-fitting facet in each pixel compared to the fit for the rest of the facets. To calculate this dominance value, we first computed the exponential of scores for Tiers 1 and 2 to scale their values to a positive range and remove the influence of high negative values, as these were not considered separately in the classification (see methods under Factor Analysis). We then calculated the difference between scores of the best and second-best facet and divided this value by the total range of scores. The resulting value is a standard measure (between 0 and 1) that represents the dominance of the strongest facet in each pixel, which reflects how it was classified. Thus, index values approaching 1 indicate that the mapped unit in a particular pixel is the only likely candidate for that pixel, and mapping is highly certain. Alternatively, a value of 0 indicates that two or more units are equally likely to occur in that pixel and mapping is highly uncertain. A useful benchmark to consider is a situation where the fit of the units increases linearly from worst to best. In this situation, a classification with six potential units would produce a dominance value of 1/(6-1) = 0.2. If the fit improves exponentially over five units, e.g. by a factor of two each time, the dominance value of the best unit would be 0.53.

In Tier 1, 80% of pixels had a dominance value of 0.2 or greater. Approximately 10% of pixels had a dominance value below 0.1. In Tier 2, 75% of pixels had a dominance of 0.2 or greater, and 11% of pixels had a dominance value below 0.1. There was no obvious biogeographical pattern in the dominance values, but they tended to be lower on the spatial borders between ecosystems and in complex configurations of ice-free patches.

## Supplementary information

Table S2

Table S1

## Data Availability

All code used to develop the data records described in this paper is available at https://github.com/anikobtoth/Antarctica.
